# Evaluation einer Therapieanpassung bei ASS-Low-Response in der Gefäßchirurgie

**DOI:** 10.1007/s00104-020-01280-x

**Published:** 2020-09-18

**Authors:** T Hummel, S. H. Meves, A. Breuer-Kaiser, J. O. Düsterwald, D. Mühlberger, A. Mumme, H. Neubauer

**Affiliations:** 1 Klinik für Gefäßchirurgie, St. Josef Hospital, Ruhr-Universität Bochum, Gudrunstraße 56, 44791 Bochum, Deutschland; 2 Klinik für Neurologie, St. Josef Hospital, Ruhr-Universität Bochum, Gudrunstraße 56, 44791 Bochum, Deutschland; 3 Klinik für Anästhesiologie und Intensivmedizin, St. Josef Hospital, Ruhr-Universität Bochum, Gudrunstraße 56, 44791 Bochum, Deutschland; 4 Klinik für Kardiologie, St. Josef Hospital, Ruhr-Universität Bochum, Gudrunstraße 56, 44791 Bochum, Deutschland

**Keywords:** High on-treatment platelet reactivity, Acetylsalicylsäure, Aspirin, Low-Response, Aggregometrie, High on-treatment platelet reactivity, Acetylsalicylic acid, Aspirin, Low response, Aggregometry

## Abstract

**Hintergrund:**

Eine verminderte antithrombozytäre Prophylaxe („Low-Response [LR]“/„high on-treatment platelet reactivity [HPR]“) mit Acetylsalicylsäure (ASS) ist mit einem erhöhten Risiko für thrombembolische Ereignisse assoziiert. Die Prävalenz einer Low-Response ist mit ca. 20 % häufig und ein Therapieregime wurde bisher noch nicht etabliert. Das Ziel dieser prospektiven Studie war es, die Effektivität eines Therapieschemas zur Therapieanpassung bei detektierter LR/HPR bei gefäßchirurgischen Patienten zu evaluieren.

**Methodik:**

Insgesamt wurden 36 gefäßchirurgischen Patienten mit einer antithrombozytären Dauermedikation mit ASS 100 mg/Tag und einer nachgewiesenen ASS-Low-Response (ALR) in die Studie eingeschlossen. Entsprechend dem festgelegten Therapieplan wurde bei diesen Patienten eine Therapieanpassung durchgeführt und eine Kontrollaggregometrie zur Erfolgskontrolle durchgeführt. Das verwendete Therapieschema folgte dem Test-and-treat-Prinzip. Zur Beurteilung der Wirkung von ASS diente die Impedanzaggregometrie mittels Mehrelektrodenaggregometer (Multiplate).

**Ergebnisse:**

Insgesamt konnten alle 36 Patienten erfolgreich in eine Response überführt werden. Bei 32 (88,89 %) Patienten erfolgte eine Dosiserhöhung auf 300 mg ASS, 2 (5,56 %) Patienten wurden von ASS auf Clopidogrel umgestellt. Bei weiteren 2 (5,56 %) Patienten wurde auf eine orale Antikoagulation mit Phenprocoumon aufgrund anderer Indikationen umgestellt. Blutungskomplikationen oder Nebenwirkungen traten nicht auf.

**Schlussfolgerung:**

Das gewählte Therapieschema zur Behandlung einer Low-Response erwies sich als effektiv und sicher bei gefäßchirurgischen Patienten. Überwiegend führte eine leitliniengerechte Dosiserhöhung der Prophylaxe von 100 mg auf 300 mg ASS zu einer effektiven Thrombozytenaggregationshemmung in der Aggregometrie.

## Abstract (en)

**Background:**

A decreased antiplatelet prophylaxis (low response, LR/high on-treatment platelet reactivity, HPR) with acetylsalicylic acid (ASA) is associated with an increased risk of thromboembolic events. The prevalence of a LR is frequent with about 20% and a therapeutic regimen is not yet established. The aim of this prospective study was to evaluate the effectiveness of a therapeutic regimen for treatment adaptation when LR/HPR is detected in vascular surgery patients.

**Methods:**

Overall, 36 patients under long-term antiplatelet treatment with 100 mg/day ASA and a detected ASA low response (ALR) were included in the study. In this patient group a modification of the prophylactic medication was carried out according to the established treatment plan and a control aggregometry was performed. The therapeutic regimen followed the test and treat principle. To evaluate the effect of ASA impedance, aggregometry with multiple electrodes was used (multiplate).

**Results:**

All 36 patients were successfully transferred to response status with the treatment scheme. In 32 (88.89%) patients an increased dose of 300 mg/day ASA was carried out and in 2 (5.56%) patients the medication was changed from ASA to clopidogrel. A further 2 (5.56%) patients were switched to oral anticoagulation with phenprocoumon, due to other indications. Bleeding complications or other side effects did not occur.

**Conclusion:**

The chosen treatment regime for a low response proved to be effective and safe in vascular surgery patients. A guideline-compliant increase of the ASA dose from 100 mg to 300 mg/day predominantly led to an effective inhibition of platelet aggregation in the aggregometry.

ASS ist nach wie vor das am häufigsten verwendete Medikament in der primären und sekundären Prophylaxe kardiovaskulärer Patienten. Allerdings gibt es eine individuelle Wirkungsschwankung der Medikation mit einer unzureichenden Thrombozytenhemmung (Low-Response) in der Aggregometrie. Wie solche „Therapieversager“ behandelt werden sollen, ist bisher nicht einheitlich geklärt. Die hier vorliegende Untersuchung wurde initiiert, um die Effektivität eines Therapieschemas im gefäßchirurgischen Patientengut zu evaluieren.

## Hintergrund

In der sekundären und tertiären Prophylaxe einer koronaren oder peripheren arteriellen Verschlusserkrankung wird Acetylsalicylsäure (ASS) oder Clopidogrel von den Fachgesellschaften zum Schutz vor thrombembolischen Ereignissen empfohlen [1, 2]. In Deutschland ist ASS das am häufigsten verordnete Medikament zur Prophylaxe kardiovaskulärer Erkrankungen. Allerdings hat sich in vornehmlich an kardiologischen Patienten durchgeführten Untersuchungen gezeigt, dass es individuelle Schwankungen in der Effektivität der antithrombozytären Wirkung gibt [3–5]. Nach Weber [6] werden drei Typen einer ASS-Resistenz unterschieden (Tab. 1).

**Table 1:** 

Typ I	*Pharmakokinetische Resistenz:* Weder die Aggregation noch die Thomboxansynthese werden nach oraler ASS-Gabe gehemmt – die In-vitro-Gabe von ASS hemmt jedoch beide
Typ II	*Pharmokodynamische Resistenz:* Hier werden sowohl nach oraler als auch nach In-vitro-Gabe von ASS weder die Aggregation noch die Thromboxansynthese gehemmt
Typ III	*Pseudoresistenz:* Hier wird sowohl nach oraler als auch nach In-vitro-Gabe von ASS die Thromboxansynthese durch thromboxanunabhängige Mechanismen gehemmt, jedoch nicht die Aggregation

Die klinische Relevanz einer Low-Response konnte durch mehrere Untersuchungen belegt werden. Hierbei wurde eine signifikante Erhöhung klinisch relevanter ischämischer kardiovaskulärer Ereignisse in der Gruppe der Low-Responder nachgewiesen [7–9].

Die Prävalenz dieser Therapieversager schwankt in der Literatur und ist in der aktuellen Diskussion bei gefäßchirurgischen Patienten unterrepräsentiert. In einer eigenen monozentrischen Prävalenzuntersuchung bei Patienten mit einer pAVK (periphere arterielle Verschlusserkrankung) oder einer Karotisstenose bestand die Prävalenz einer sog. „high on-treatment platelet reactivity“ (HPR, Low-Response [LR]) für ASS (ALR) bei 19,3 % und für Clopidogrel (CLR) bei 21,1 % der mittels Impedanzaggregometrie untersuchten Patienten. Jeder fünfte unserer Patienten hatte somit bereits vor gefäßtherapeutischen Maßnahmen keine wirksame antithrombozytäre Prophylaxe [10]. Wie allerdings mit einer solchen ineffektiven Medikation umzugehen ist und welche therapeutischen Maßnahmen bei solchen Patienten zu treffen sind, ist bisher nicht einheitlich geregelt. Die bestehenden Leitlinien geben hier in der Prophylaxe einen gewissen therapeutischen Spielraum. So wird für die prophylaktische Standardtherapie der pAVK mit ASS in Deutschland eine Dosierung von 75–300 mg empfohlen [2].

Aufgrund der hohen klinischen Relevanz einer ineffektiven thrombembolischen Prophylaxe wurde im Rahmen dieser Studie ein Therapieschema zur Behandlung einer detektierten ALR an gefäßchirurgischen Patienten evaluiert.

## Material und Methoden

Eingeschlossen in die Studie wurden gefäßchirurgische Routinepatienten mit einer symptomatischen pAVK oder einer behandlungsbedürftigen A.-carotis interna-Stenose, bei denen eine elektive invasive Gefäßdiagnostik oder eine operative bzw. interventionelle Therapie unter einer laufenden antithrombozytären Dauertherapie mit ASS 100 mg durchgeführt wurde. Um eine Noncompliance oder Unregelmäßigkeiten in der Dauermedikation auszuschließen, erfolgt eine intensive Befragung der Patienten bezüglich der konsequenten täglichen Einnahme der Medikamente und der Darreichungsroutine innerhalb der letzten 14 Tage vor Studieneinschluss. Erfasst wurden neben den demographischen Daten die Begleiterkrankungen, die Nebenmedikation und Laborparameter zum Ausschluss anderer Ursachen der Low-Response der antithrombozytären Therapie. Um den Schweregrad der arteriosklerotischen Gefäßveränderung abschätzen zu können, wurde die Lokalisation dokumentiert und der Schweregrad der pAVK mittels der Stadieneinteilung nach Fontaine vorgenommen.

Ausschlusskriterien waren neben einer Nonadhärenz zur Medikation oder Unregelmäßigkeiten in der Einnahme die Einnahme einer ASS-„Loading-dose“ innerhalb der letzten 14 Tage vor Studieneinschluss, eine abnormal erhöhte oder verminderte Thrombozytenzahl, schwere internistische Begleiterkrankungen wie kürzlich stattgehabte gastrointestinale Blutungen, maligne Erkrankungen, schwergradige Herz- oder Leberinsuffizienz und bekannte Gerinnungsstörungen, um eine intrinsische Aktivierung oder Deprimierung der Thrombozytenaktivität auszuschließen.

Die Blutentnahme erfolgte nach entsprechender Aufklärung und Einverständniserklärung des Patienten mit einer 21-G-Kanüle aus einer Kubitalvene. Hierbei wurde der Kolben der Monovetten kontinuierlich und nicht ruckartig zurückgezogen, um Scherstress der Thrombozyten zu verhindern. Nach der Punktion wurden die ersten 4 ml Vollblut verworfen, um eine spontane Aktivierung der Thrombozyten zu vermeiden. Zur Bestimmung dienten 4‑ml-Röhrchen mit Natriumzitrat sowie 2,7-ml-Röhrchen mit r‑Hirudin als Antikoagulans. Um die Refraktärzeit der Thrombozyten nach der Blutentnahme abzuwarten [11, 12], wurden die entnommenen Blutproben bis zur Verarbeitung mindestens 30 min bei Raumtemperatur gelagert. Alle Blutanalysen wurden innerhalb von 3 h nach Blutentnahme durchgeführt, da in dieser Zeitspanne die Messergebnisse am konstantesten sind [13].

Um den plättchenhemmenden Effekt von ASS zu überprüfen, erfolgte eine Vollblutaggregometrie mittels Mehrelektrodenimpedanzaggregometer („Multiple Electrode Aggregometry“/Multiplate®-Analyzer, Roche, Schweiz), bei der mit zwei Messelektrodenpaaren gleichzeitig gemessen wird. Eine Abweichung der einzelnen Messergebnisse von mehr als 20 % vom Mittelwert oder einem Korrelationskoeffizienten zwischen beiden Messwerten unterhalb von 0,98 wurde als fehlerhaft gewertet und die Messung wiederholt. Die Auswertung der Messergebnisse erfolgte entsprechend den Herstellerangaben und den bereits in der Literatur beschriebenen Methoden für ASS [14–17]. Für die Messungen der Aggregationsfähigkeit unter ASS wurde die ASPI-Lösung (Arachidonsäure) benutzt. Diese bewirkt eine Thrombozytenaktivierung durch Arachidonsäure, welche über die Zyklooxygenase (COX) zusammen mit der Thromboxansynthase in Thromboxan-A2 umgewandelt wird. Entsprechend den Herstellerempfehlungen und vorherigen Untersuchungen wurde der Cut-off-Wert für ALR bei >46 U verwendet [18, 19].

**Figure 1:**
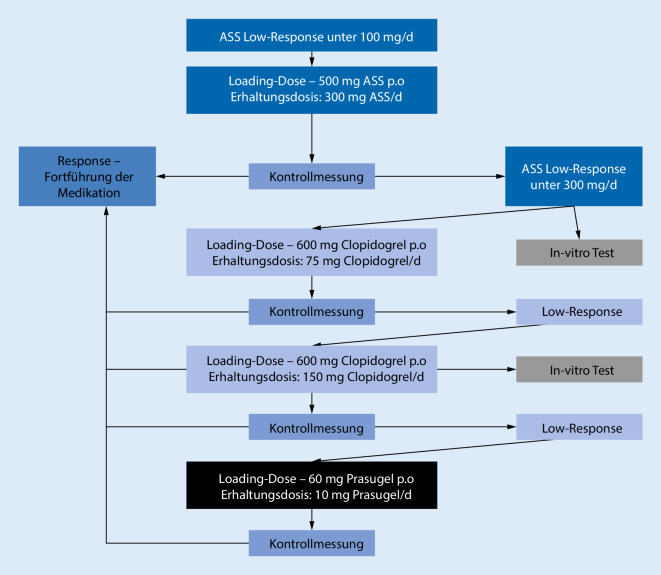

Bei detektierter Low-Response erfolgte eine Therapieanpassung nach der gefäßchirurgischen Intervention gemäß dem zuvor festgelegten Therapieschema nach dem Test-and-treat-Prinzip. Hierbei wurde bei festgestellter ALR eine „loading dose“ von 500 mg ASS per os gefolgt von einer Erhaltungsdosis von 300 mg ASS/Tag verabreicht. Nach 48 h wurde eine Kontrollmessung durchgeführt. Bei erfolgreicher Therapieanpassung wurde die Erhaltungsdosis bei 300 mg ASS/Tag belassen. Bei einer erneuten LR unter 300 mg ASS wurde ein In-vitro-Test zur Differenzierung einer pharmakokinetischen oder -dynamischen Ätiologie der LR entsprechend der Typeneinteilung nach Weber durchgeführt (siehe Tab. 1). Bei einer persistenten ALR erfolgte eine Umstellung der Medikation von ASS auf Clopidogrel. Nach einer „loading dose“ von 600 mg Clopidogrel und einer Erhaltungsdosis von 75 mg/Tag erfolgte nach 48 h eine Kontrollmessung. War nun eine regelrechte Thrombozytenhemmung nachweisbar, wurde die Medikation mit 75 mg Clopidogrel auf Dauer fortgesetzt. Bei einer in der Kontrollmessung neu detektierten Low-Response für Clopidogrel (CLR) wurde eine erneute „loading dose“ von 600 mg gegeben und die Erhaltungsdosis auf 2‑mal 75 mg/Tag erhöht. Zusätzlich wurde bei CLR eine In-vitro-Messung zur Überprüfung der Funktionsfähigkeit des ADP-Rezeptors durchgeführt. Eine erneute Kontrollmessung fand nach 48 h statt. Falls auch hierunter eine CLR zu verzeichnen war, erfolgte eine erneute Umstellung der Medikation nach einer „loading dose“ von 60 mg Prasugrel auf eine Erhaltungsdosis von 10 mg Prasugrel/Tag. Abb. 1 zeigt das Schema zur Therapieanpassung bei einer ALR.

Die Studie wurde von der zuständigen Ethikkommission der Ruhr-Universität Bochum geprüft und bewilligt und ist im Einklang mit nationalem Recht sowie gemäß der Deklaration von Helsinki von 1975 (in der aktuellen, überarbeiteten Fassung) durchgeführt worden. Von allen beteiligten Patienten liegt eine Einverständniserklärung vor.

Die Datensammlung und deskriptive Statistik erfolgte mittels Microsoft Excel (Version 14.6.4, Microsoft Corporation).

## Ergebnisse

Insgesamt 36 Patienten mit einer nachgewiesenen ASS-Low-Response wurden in die Studie eingeschlossen. 22 (61,11 %) Patienten hatten als Aufnahmegrund eine symptomatische pAVK und 14 (38,89 %) eine hochgradige A.-carotis-interna-Stenose. Die demographischen Daten, den Schweregrad der Erkrankung und den im Rahmen des stationären Aufenthaltes durchgeführten Eingriff beschreibt Tab. 2.

**Table 2:** 

Total	*n* = 36
Durchschnittsalter in Jahren (SD)	76,61 (±11,40)
BMI kg/m^2^ (SD)	27,02 (±4,99)
Männlich (%)	20 (55,56)
Weiblich (%)	16 (44,44)
*Aufnahmegrund*	
Karotisstenose (%)	14 (38,89)
*(p)AVK (%)*	22 (61,11)
*pAVK Stadium nach Fontain*	
Stadium 1 (%)	0 (0)
Stadium 2 (%)	12 (33,33)
Stadium 3 (%)	4 (11,11)
Stadium 4 (%)	6 (16,67)
*Klinische Einteilung der Karotisstenose*	
Asymptomatisch (%)	9 (25,00)
Symptomatisch (%)	5 (13,89)
*Durchgeführte Maßnahme*	
Stent/PTA (%)	6 (16,67)
Diagnostische Angiographie (%)	4 (11,11)
Bypassanlage (%)	8 (22,22)
Femoralis-TEA (%)	4 (11,11)
Karotis-TEA (%)	14 (38,89)

*SD* Standardabweichung, *BMI* Body-Mass-Index, *PTA* perkutane transluminale Angioplastie, *TEA* Thrombendarteriektomie

Bei den Begleiterkrankungen und Risikofaktoren der pAVK waren der arterielle Hypertonus bei 25 (69,44 %) Patienten und der Nikotinabusus bei 24 (66,67 %) der Patienten führend. Im Durchschnitt nahmen die Patienten 5,18 (±3,03) Medikamente neben der ASS Prophylaxe ein (vergleiche hierzu Tab. 3).

**Table 3:** 

Total	*n* = 36
*Begleiterkrankungen*	
Arterielle Hypertonie (%)	25 (69,44)
Diabetes mellitus (%)	10 (27,78)
Rauchen (%)	24 (66,67)
Hypercholesterinämie (%)	5 (13,89)
Apoplex (%)	5 (13,89)
KHK (%)	9 (25,00)
*Komedikation*	
ACE-Inhibitor/AT1 (%)	21 (58,33)
β‑Blocker (%)	16 (44,44)
Ca-Kanal-Blocker (%)	9 (25,00)
Diuretika (%)	13 (36,11)
Orale Antidiabetika (%)	4 (11,11)
Insulin (%)	5 (13,89)
Nitrate (%)	4 (11,11)
Statine (%)	18 (50,00)
Protonenpumpeninhibitor (%)	9 (25,00)
Pantoprazol (%)	7 (19,44)
Omeprazol/Esomeprazol (%)	1 (2,78)
Antidepressiva (%)	4 (11,11)
Anzahl Komedikation (SD)	5,18 (±3,03)
*Laborparameter*	
Leukozyten (x10^9^/l) (SD)	8681,14 (±2400,42)
Hämoglobin (g/dl) (SD)	13.22 (±2,24)
Thrombozyten (/µl) (SD)	273314,29 (±99265,24)
Kreatinin (µmol/l) (SD)	1.15 (±0.89)
GFR (ml/min) (SD)	72,51 (±29,08)
HbA1_c_ (%) (SD)	6.13 (±1,18)
Gesamtcholesterin (SD)	204,92 (±43.43)
LDL/HDL (SD)	2.55 (±1,05)
CRP (mg/l) (SD)	16,00 (±31,67)

Anhand des in Abb. 1 dargestellten Therapieplans wurde die Umstellung der Medikation durchgeführt, um eine wirksame Thrombozytenaggregationshemmung zu erzielen. Bei 32 (88,89 %) der Patienten war bereits der erste Schritt des Therapieplans mit der Gabe einer „loading dose“ von 500 mg ASS oral und anschließender erhöhter Erhaltungsdosis von 300 mg ASS täglich erfolgreich. Hier war in der Aggregometrie eine effektive Thrombozytenaggregationshemmung nach 48 h messbar.

Zwei (5,56 %) Patienten wiesen eine persistente ALR in der Kontrolle auf, sodass eine Umstellung der Medikation auf Clopidogrel erfolgte. Hierbei zeigte sich, nach Gabe einer „loading dose“ von 600 mg Clopidogrel mit Erhaltungsdosis von 75 mg/Tag Clopidogrel, ein effektives Therapieergebnis in der Aggregometrie.

**Figure 2:**
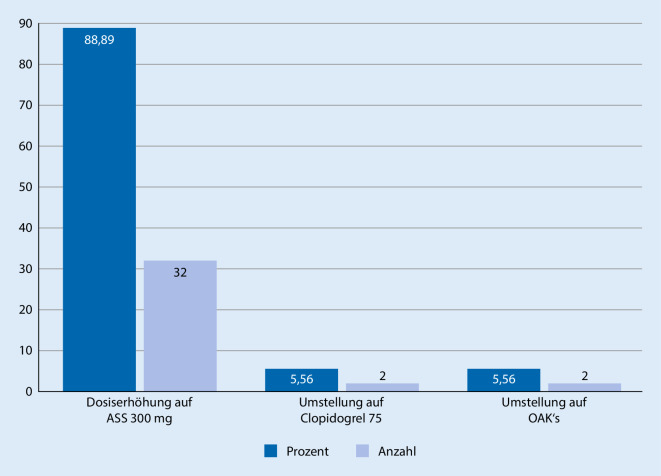

Bei 2 (5,56 %) Patienten wurde aufgrund eines neu aufgetretenen Vorhofflimmerns und aufgrund einer peripheren Revaskularisation bei schlechter Ausstrombahn die antithrombozytäre Prophylaxe mit ASS 100 mg auf eine orale Antikoagulation mit Phenprocoumon umgestellt, sodass eine weitere Therapieanpassung nicht vorgenommen wurde. Abb. 2 zeigt die vorgenommenen Therapieanpassungen.

## Diskussion

Die vorliegende Studie konnte zeigen, dass durch den von uns angewandten Therapiealgorithmus bei allen Patienten mit einer nachgewiesenen Low-Response eine suffiziente Thrombozytenaggregation erreicht werden konnte. Hierbei wurde effektiv bei 89 % des Patientenkollektivs die ASS-Dosis von 100 mg nach einmaliger „loading dose“ von 500 mg/Tag auf 300 mg/Tag ASS erhöht. Diese Anpassungsdosis bewegt sich innerhalb der empfohlenen und zugelassenen Tagesdosis der aktuellen Leitlinien, da diese zwischen 75 mg und 300 mg ASS/Tag als sekundäre und tertiäre Prophylaxe nach gefäßchirurgischen Eingriffen liegt [2]. Andere Untersuchungen konnten ebenfalls eine Korrelation einer einfachen Dosiserhöhung mit einer Reduktion der Low-Response-Rate nachweisen. Hovens et al. zeigten in einer Metaanalyse eine signifikant höhere Prävalenz der ALR von 36 % unter ≤100 mg/Tag im Vergleich zu Patienten mit einer ASS-Medikation mit 300 mg/Tag, welche bei 19 % lag [4]. Neubauer et al. konnten bei 504 Patienten mit einer koronaren Herzkrankheit eine ALR von 19,8 % nachweisen. Nach einer Dosiserhöhung auf 300 mg ASS zeigte sich ebenfalls eine Reduktion der Low-Response-Rate um 94,6 %. Nach einer Gabe von 500 mg ASS als Dauermedikation zeigten auch die übrigen Patienten in der Studie eine Response [20]. Gonzales-Conejero et al. demonstrierten eine ALR-Prävalenzreduktion nach einmaliger Gabe von 500 mg ASS per os und ten Berg et al. nach einer einmaligen Bolusinjektion von 1000 mg ASS [21, 22].

Frelinger et al. beschrieben, dass eine ASS-LR durch eine zu geringe Dosierung oder Complianceproblematik bedingt sein kann. Dies schlussfolgerten die Autoren, da sie durch eine Ex-vivo-Zugabe von ASS einen antithrombozytären Effekt nachweisen konnten [3]. In unserer Studie zeigten ebenfalls alle Patienten nach Inkubation der Blutprobe in vitro einen ausreichenden thrombozytenfunktionshemmenden Effekt, sodass von einer pharmakokinetischen Ursache ausgegangen werden kann und nicht von einem Rezeptordefekt. Ob die Ergebnisse dieser Studie, trotz eingehender Medikamentenanamnese, durch eine medikamentöse Noncompliance beeinflusst wurden, bleibt weiterhin unklar. Als Ursache für eine Low-Response wird die fehlende Medikamentenadhärenz mit 9 % aller Fälle in der Literatur angegeben [23].

In der Bewertung der Therapieoptimierung wurden keine durch die Anpassung der Medikation assoziierten Komplikationen (Blutungen, gastrointestinale Komplikationen oder Medikamentennebenwirkungen) registriert. Allerdings konnten einige Studien nachweisen, dass mit steigender ASS-Dosierung ein erhöhtes Blutungsrisiko besteht und darüber hinaus eine niedrige ASS-Dosis den gleichen Effekt erzielt wie eine hohe ASS-Dosis [24, 25]. Demgegenüber steht die CURRENT-OASIS-7-Studie, die keinen signifikanten Unterschied zwischen niedriger Dosierung (75–100 mg ASS) und höherer Dosierung (300–325 mg ASS) bezogen auf Blutungskomplikationen nachweisen konnte [26].

In der vorliegenden Studie wurden 2 Patienten entsprechend dem Therapieschema von ASS auf Clopidogrel 75 mg/Tag erfolgreich umgestellt. Bei keinem der Patienten war eine Umstellung auf Prasugrel notwendig.

Prasugrel wurde in das Therapieregime als „Bail-out“-Lösung für eine persistente Low-Response auf die etablierten Medikamente aufgenommen. Die Effektivität einer Umstellung bei bestehender CLR auf Prasugrel zeigten Campo et al. in einer Cross-over-Studie an 143 Patienten [27]. Ebenso konnten Weerakkody et al. in einer Untersuchung an 131 Patienten eine geringere Prävalenz einer Low-Response von Prasugrel im Vergleich zu Clopidogrel nachweisen [28]. In einer Untersuchung von Wiviott et al. bestand ebenfalls eine geringerer Rate ischämischer Ereignisse unter Prasugrel im Vergleich zu Clopidogrel, allerdings ging dieser Vorteil mit einer höheren Blutungsrate bei identischer Gesamtmortalität einher [29]. Die Gefahr einer vermehrten Blutungskomplikation muss im Einzelfall abgewogen werden und kann anhand der in unserer Studie gewonnen Daten nicht bewertet werden.

## Limitationen und Ausblicke

Im Rahmen dieser Untersuchung ließen sich alle ASS-Low-Responder erfolgreich und leitliniengerecht anpassen, sei es durch eine Dosiserhöhung auf 300 mg/Tag ASS oder durch eine Umstellung des Präparates auf Clopidogrel. Insgesamt erwies sich das gewählte Therapieschema als effektiv und sicher. Aufgrund der im Vergleich geringen Fallzahl und des gewählten einseitigen Testprinzips mittels Impedanzaggregometrie bleibt die Aussagekraft dieser Studie beschränkt. Ob eine Eskalation der Therapieumstellung auf Prasugrel ein erhöhtes Blutungsrisiko für gefäßchirurgische Patienten darstellt oder zu einer effektiven Prophylaxe in diesem Patientengut führt, kann anhand dieser Untersuchung nicht bewertet werden, da es bei keinem Patienten notwendig war, die Medikation auf Prasugrel zu ändern. Im Einzelfall muss vor einer Umstellung der Medikation eine Nutzen-Risiko-Abwägung hinsichtlich des individuellen Blutungsrisikos und des gefäßchirurgischen Eingriffes erfolgen. Ob die Therapieumstellung im Test-and-treat-Prinzip auch zu besseren klinischen Ergebnissen in der Prophylaxe thrombembolischer Ereignisse führt, müssen weitere Studien mit klinischem Follow-up nachweisen. In Bezug auf eine optimale Dosisfindung in einer Nutzen-Risiko-Evaluation der ASS-Prophylaxe werden mit Spannung die ersten Ergebnisse der US-amerikanischen prospektiven ADAPTABLE-Studie (Aspirin Dosing: A Patient-centric Trial Assessing Benefits and Long-term Effectiveness) erwartet [30]. Hier werden die in den USA erhältlichen Dosierungen von 81 und 325 mg ASS in der sekundären Prophylaxe miteinander verglichen. 2019 ist der Patienteneinschluss mit 15.000 Probanden abgeschlossen worden. Erste Ergebnisse sind für das Jahr 2020 angekündigt worden. Eventuell muss dann die bisher praktizierte „One-size-fits-all“-Strategie noch einmal überdacht werden. Hinsichtlich der Detektion von Low-Respondern kann eine generelle Aggregometrie der gefäßchirurgischen Patienten in einer Kosten-Nutzen-Relation unserer Meinung nach nicht empfohlen werden, wir sind in der klinischen Praxis zu einer Testung bei auffälligem klinischem Verlauf übergegangen.

## Fazit für die Praxis

- Etwa jeder 5. Patient mit einer Acetylsalicylsäure(ASS)-Prophylaxe hat eine ineffektive Thrombozytenaggregationshemmung in der Aggregometrie.
- Vor einer Therapieanpassung bei vorliegender Low-Response muss zunächst eine medikamentöse Noncompliance ausgeschlossen werden.
- Das in der Studie evaluierte Therapiekonzept zur medikamentösen Therapie der ASS-Low-Response erwies sich als effektiv und sicher.
- In ca. 90 % der Fälle führte eine leitliniengerechte Dosiserhöhung auf 300 mg/Tag ASS zu einer effektiven Thrombozytenaggregationshemmung in der Impedanzaggregometrie.
